# Raspberry Ketones Attenuate Cyclophosphamide-Induced Pulmonary Toxicity in Mice through Inhibition of Oxidative Stress and NF-ΚB Pathway

**DOI:** 10.3390/antiox9111168

**Published:** 2020-11-23

**Authors:** Marwa T. Mohamed, Sawsan A. Zaitone, Amal Ahmed, Eman T. Mehanna, Norhan M. El-Sayed

**Affiliations:** 1 Directorate of Health and population, Ismailia 41522, Egypt; marwa_tarekmoh@pharm.suez.edu.eg; 2 Department of Pharmacology and Toxicology, Faculty of Pharmacy, Suez Canal University, Ismailia 41522, Egypt; sawsan_zaytoon@pharm.suez.edu.eg; 3 Department of Pharmacology and Toxicology, Faculty of Pharmacy, University of Tabuk, Tabuk 741, Saudi Arabia; 4 Department of Cytology and Histology, Faculty of Veterinary Medicine, Suez Canal University, Ismailia 41522, Egypt; amal_ahmed@vet.suez.edu.eg; 5 Department of Biochemistry, Faculty of Pharmacy, Suez Canal University, Ismailia 41522, Egypt; Eman.Taha@pharm.suez.edu.eg

**Keywords:** cyclophosphamide, raspberry ketones, oxidative stress, inflammatory pathway, pulmonary toxicity

## Abstract

Cyclophosphamide (CP) was found to have a potential toxic effect on lung tissues. Raspberry ketones (RKs) are natural antioxidant chemicals isolated from red raspberries (Rubus ideaus). They are commonly used for weight loss and obesity. The current study aimed to evaluate the possible protective effects of RKs against lung toxicity induced by CP. Mice were allocated into six groups: (1) control group; (2) CP group: received a single intraperitoneal dose of CP (150 mg/kg, i.p.); and (3–6) mice were pre-treated orally with different doses of RKs (25, 50, 100, and 200 mg/kg) for 14 consecutive days, respectively, before the administration of an intraperitoneal dose of CP (150 mg/kg, i.p.). Mice were then sacrificed under anesthesia, then lungs were removed for histopathological and biochemical investigations. A single dose of CP markedly altered the levels of some oxidative stress biomarkers and resulted in the fragmentation of DNA in lung homogenates. Histological examination of CP-treated mice demonstrated diffuse alveolar damage that involved apparent hyalinization of membranes, thickening of inter alveolar septa, and proliferation of type II pneumocytes. The immunohistochemical results of CP-treated mice revealed strongly positive Bax and weakly positive proliferating cell nuclear antigen (PCNA) staining reactivity of the nuclei of the lining epithelium of the bronchioles and alveoli. CP activated the cyclooxygenase-2/nuclear factor-kappa B pathway. However, pre-treatment with RKs significantly attenuated CP-evoked alterations in the previously mentioned parameters, highlighting their antioxidant, anti-inflammatory, and anti-apoptotic potential. RKs may be suggested to be a potential candidate to ameliorate CP-induced pulmonary toxicity.

## 1. Introduction

Cyclophosphamide (CP) is a chemotherapeutic agent that is commonly used in the treatment of a variety of human neoplasia and as immunosuppressive therapy following organ transplants. It is also used in some autoimmune disorders such as nephritic syndrome in children, Wegener’s granulomatosis, and rheumatoid arthritis [1,2]. CP is metabolized by a microsomal cytochrome P450 enzyme in the liver into phosphoramide mustard and acrolein. The latter is related to CP’s side effects via inhibition of the endogenous antioxidant system and enhancing the production of reactive oxygen species (ROS) in the cells [3]. These free radicals damage the DNA, leading to a variety of pathological findings [4].

Exposure to CP also augments inflammation with the association of action of nuclear factor-kappa B (NF-KB) [5]. CP has been shown to cause acute and chronic pulmonary injury in both humans and animals [6]. A characteristic histopathologic finding detected in patients with CP pulmonary toxicity is the presence of atypical cells in the alveolar and bronchiolar epithelium, besides the thickening of type II pneumocytes, leading to interstitial and alveolar oedema and fibrosis [7]. CP was also demonstrated to promote pulmonary tumour progression, and hence metastasis by the synergistic effect of matrix degrading proteases and adhesion proteins [8].

Clinically, pulmonary side effects of CP manifest as either the early phase of pneumonitis with cases presenting cough, dyspnoea occurring within the first six months of starting CP therapy or late onset fibrosis with progressive pulmonary fibrosis, and bilateral pleural thickening [9]. Therefore, there is a dire need for an agent that is capable of quenching or scavenging the toxic effect of CP metabolites without affecting the therapeutic effect of CP as anti-tumour or immunosuppressant.

Raspberry ketones (RKs) are major aromatic compounds obtained from red raspberry (Rubus ideaus). The chemical structures of RKs are 4-(4-hydroxyphenyl)butan-2one, similar to the structures of capsaicin, and synephrine, which are responsible for its anti-obesity actions and alteration of lipid metabolism [10]. RKs demonstrated antioxidant effects through the reduction of abnormally high levels of malondialdehyde (MDA) [11]. Moreover, RKs were reported to have an anti-inflammatory activity by blocking the NF-KB pathway. They prevented the activation of NF-KB by blocking the translocation of NF-KB into the nucleus by IkB degeneration [12].

Exposure to CP can increase the risk of pulmonary toxicity assigned by biochemical and histopathological changes. The current study aimed to investigate the possible protective effect of RKs against lung toxicity induced by CP in mice.

## 2. Materials and Methods

### 2.1. Chemicals

Raspberry ketones were purchased from (ABSONUTRIX, 5500-206 Adams Farm Lane, Greensboro, NC 27407, USA). They were dissolved in sesame oil (Ever Line, Egypt). Cyclophosphamide (Endoxan) was provided by Baxter Oncology GMBH (Westfalen, Germany).

### 2.2. Animals

Forty-eight male Swiss albino mice weighting 20–30 grams were used. They were obtained from the animal house of the national research center, Dokki, Giza, Egypt. Mice were housed in a controlled temperature room at 25 ± 1 °C under standard conditions on 12 h dark and light cycles. All experimental procedures were conducted in accordance with the Guidelines of the Animal Care and use Committee at the Faculty of Pharmacy, Suez Canal University (approval no. 201704RA2).

### 2.3. Experimental Design

Mice were randomly distributed into six groups, each containing eight animals.

Group 1 (the vehicle control group): the mice received a single dose of saline (10 mL/kg b.w., i.p.) and sesame oil (10 mL/kg b.w.) by oral gavage for 14 days.

Group 2: mice administered a single toxic dose of CP (150 mg/kg, i.p.) [13] diluted in normal saline (10 mL/kg b.w.) and sesame oil (10 mL/kg b.w.) by oral gavage for 14 days.

Groups 3–6: mice were treated with RKs at different concentrations (25, 50, 100, and 200 mg/kg b.w, p.o.) [14] dissolved in sesame oil (10 mL/kg b.w.) each day for 14 consecutive days. On the 14th day, a single toxic dose of CP (150 mg/kg, i.p.) diluted in normal saline (10 mL/kg, b.w.) was given to all groups taking RKs just 1 h after the last dose of RKs.

After 24 h of CP injection, all mice were anesthetized by ketamine dose (40 mg/kg, i.p.). Lungs were then removed and washed three times with normal saline for complete blood removal. The right lungs were dissected and used for histopathological staining and immunohistochemistry. The left lung was frozen, stored at −80 °C, and used subsequently for biochemical analysis.

### 2.4. Histopathology and Immunohistochemistry

#### 2.4.1. Histopathological Evaluation

The right lung lobes were cut into small pieces, fixed in 10% neutral buffered formalin. Tissues were subjected to dehydration, clearing, embedding in paraffin wax, and sectioning at 5–7 μm using a Leitz 1512 microtome. Then, staining procedures were conducted by Harris’s haematoxylin and eosin (H&E) stain for light microscopic evaluation [15]. Thickening of alveolar septa, peribranchial, perivascular, congestion or haemorrhage, and infiltration by inflammatory cells were measured using a semi-quantitative method. The level of damage was based on a graded scale of 0–4, where grade 0 = no damage, 1 = slight focal damage, 2 = mild focal, 3 = moderate, and 4 = marked and severe.

They were inspected by a blinded investigator lacking knowledge of any other data on the experimental groups. All images were captured using standard digital microscope camera Olympus CX 41 binocular microscope.

#### 2.4.2. Immunohistochemistry of Bax and PCNA

For immunostaining of Bax and PCNA, lung sections (5 μm thick) were deparaffinized and heated in 0.01 mol/L citrate buffer solution (pH = 6.0) for 15 min in a microwave oven for antigen retrieval. The activity of endogenous peroxidases was quenched by applying 0.3% H_2_O_2_ to the sections. A Vectastain rabbit blocking reagent was used to prevent nonspecific binding. Polyclonal rabbit anti-Bax (Cat # ab7977, Abcam, Cambridge Science Park, Cambridge, UK) was used as primary antibodies or Mouse monoclonal, anti-PCNA (Cat # GTX100539, Thermo Scientific, CA, USA) was used as primary antibodies. Antibody bindings were visualized using avidin–biotin complex (ABC kit, Vector laboratories). Then, sections were conjugated, 3, 3-diaminobenzidine was used as a chromogen to visualize the immunoreaction, and Mayer’s haematoxylin was used for counterstaining. All procedures were done according to Vectastain Elite ABC Kit (Rabbit IgG, catalogue number PK-601, Vector Laboratories, Burlingame, CA, USA). For detection of the % area of apoptotic and proliferation cells immunostained with Bax and PCNA, respectively, images of the selected parts of lungs were analyzed using the ImageJ software developed by the National Institute of Health (Bethesda, MA, USA). Images were obtained from the lungs by means of a digital camera (Olympus Dp25, Japan).

### 2.5. Determination of Oxidative Stress Markers

Lung tissues were homogenized in ice-cold phosphate buffer, pH 7.4. The supernatants were used for determination of malondialdehyde (MDA) in addition to catalase and superoxide dismutase (SOD) activities using colorimetric kits from Bio diagnostic (Giza, Egypt) according to the manufacturer’s protocol.

### 2.6. Western Blot Analysis 

The frozen lung tissues were lysed with a Radioimmunoprecipitation assay buffer (RIPA buffer) and centrifuged at 12,000× *g* at 4 °C for 10 min to obtain the cellular proteins in the supernatant. The supernatant of lung tissues was separated by Polyacrylamide Gel Electrophoresis (SDS-PAGE), transferred to a polyvinylidene difluoride membrane, and blocked in blocking buffer (150 mM NaCl in 10 mM Tris, pH 7.5 containing 5% non-fat dry milk) for 1 h at room temperature. The membranes were incubated with primary antibodies for 18 h at 4 °C, after which they were washed three times (20 mM Tris-HCl, pH 7.5, 137 mM NaCl, and 0.1% Tween 20), incubated with horse-radish peroxidase-conjugated secondary antibodies (1:5000, Thermo Fisher Scientific, Waltham, MA, USA) for 1 h at room temperature, washed three times, and then detected with the enhanced chemiluminescence method. Antibodies against NF-KB (Cat# 8242S), cyclooxaganse-2 (COX-2) enzyme (Cat# 12282), and inducible nitric oxide synthase (iNOS) (Cat# 13120) were purchased from Cell Signaling Technology.

### 2.7. DNA Extraction and DNA Ladder by Gel Electrophoresis

Endogenous endonucleases generate mono and oligonucleosides of 180 bp or multiples among the characteristics of apoptosis. To assess the endonuclease dependent ladder like DNA fragmentation by gel electrophoresis, genomic DNA was extracted from the lung tissue using Bio EZ-10 spin column genomic DNA Kit (Markham, Canada), according to the manufacturer’s instructions. All of the extracted genomic DNA samples were diluted to 90 ng/mL, and then subjected to electrophoresis using 0.8% (*w*/*v*) agarose gel at 90 V and 110 mA for 2 h.

The samples were further visualized under UV light after ethidium bromide staining. The 100 bp DNA ladder (Solis Biodyn, Tartu, Estonia) was a ready-to-use molecular weight marker. Gel photo was captured using gel documentation system then was analyzed by Gel Docu advanced ver.2 software.

### 2.8. Statistical Analysis

The results were presented as the mean ± SE. Comparison between groups was performed applying one-way analysis of variance (ANOVA) followed by Bonferroni’s post-hoc test. The statistical analyses performed using the SPSS 23.0 software for Mac OS (SPSS Inc.©/IBM© Chicago, IL, USA) were considered significant. Data were subjected to outliers’ detections and normality testing to detect whether the data are parametric or nonparametric using the SPSS normality test. Kruskal–Wallis testing was performed to compare the groups according to distribution, as previously assessed by the Kolmogorov–Smirnov normality test. Pairwise comparison post-hoc tests were performed and the level of significance was set at *p*-values < 0.05.

## 3. Results

### 3.1. Histopathological Fndings in the Pulmonary Tissue of the Experimental Groups

**Figure 1:**
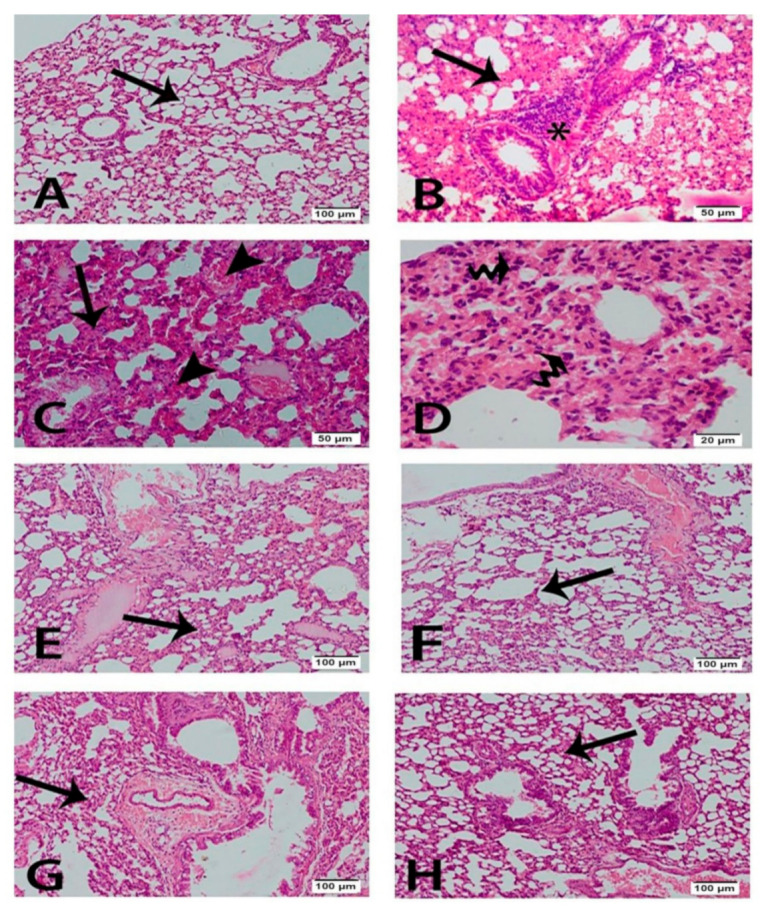
Photomicrographs of the pulmonary sections stained with H&E of different experimental groups. (**A**) Control group showed normal lung tissues with thin interalveolar septa. (**B**–**D**) CP group showing the inflammatory cell infiltrates, interalveolar thickness, (*) hyperplastic pneumocyte type II and congestion or hemorrhage. (**E**) RKs (25 mg/kg/day) and (**F**) RKs (50 mg/kg/day) treated groups showing reduction of the interalveolar thickness and inflammatory cells. (**G**) RKs (100 mg/kg/day) and (**H**) RKs (200 mg/kg/day) treated groups showing amelioration of lung tissue lesions. Notice the decrease of hyperplastic pneumocyte type II improving of the pulmonary congestion and hemorrhage. (**A**,**B**,**E**–**H**) scale bar 100 µm (**C**) scale bar 50 µm (**D**) scale bar 20 µm.

**Figure 2:**
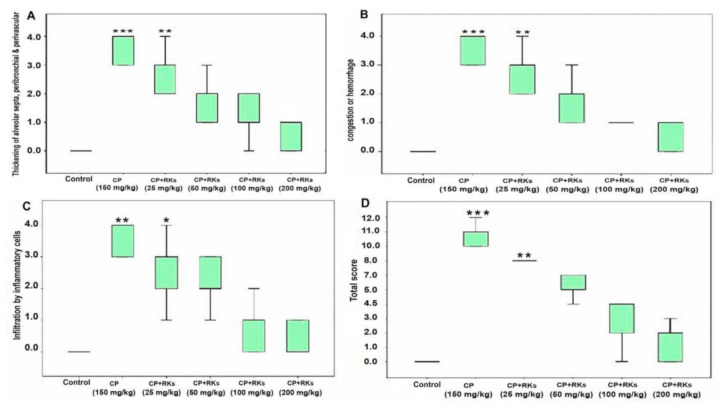
Boxplots showing scores of (**A**) thickening of alveolar septa, peribranchial and perivascular, (**B**) scores of congestion or hemorrhages, (**C**) scores of infiltrations by inflammatory cells and (**D**) the total scores of thickening (of alveolar septa, peribranchial and perivascular). All boxplot graphs treatment with different concentrations of RKs (25, 50, 100, 200 mg/kg/p.o.). Difference between groups was assessed using independent samples Kruskal-Wallis Test, followed by Kruskal-Wallis pairwise comparisons (*n* = 4–6). Asterisks denotes significant different versus CP (150 mg/Kg) (*) significant at *p* < 0.05; (**) highly significant at *p* < 0.01; (***) very high significant at *p* < 0.001).

Histological inspection of lung sections of the control group stained with H&E displayed normal histological architecture of the air conducting (intrapulmonary bronchi, and bronchioles) and respiratory portion (alveoli) of the lung. The CP-treated group showed interstitial, alveolar oedema, hemorrhage, congested blood vessels, and hyaline membranes. In addition, thickened interalveolar septa, hyperplastic pneumocyte type II, and inflammatory cell infiltration were observed. CP-induced injuries were ameliorated by the administration of different doses of RKs; the most effective enhancement was exerted using RKs at a dose of 200 mg/kg/day (Figure 1). A dramatic decrease in the thickening of interalveolar septa and inflammatory cell infiltration was observed in all RKs sections. Sections of the RKs (25 mg/kg/day) and (50 mg/kg/day) treated groups showed decreased septal alveolar thickness and inflammatory cell infiltration. Sections of the RKs (100 mg/kg/day) and (200 mg/kg/day) treated groups displayed nearly normal thickness of the interalveolar septa with the absence of inflammatory cell infiltrate (Figure 2). Congestion of the blood vessels was reduced, but there was a little interstitial hemorrhage. Furthermore, the number of the hyperplastic pneumocyte type II was gradually diminished. Moreover, the absence of the hyaline membranes was detected in the RKs pretreated groups. 

### 3.2. Immunohistochemical Fndings in the Pulmonary Tissue of the Experimental Groups

**Figure 3:**
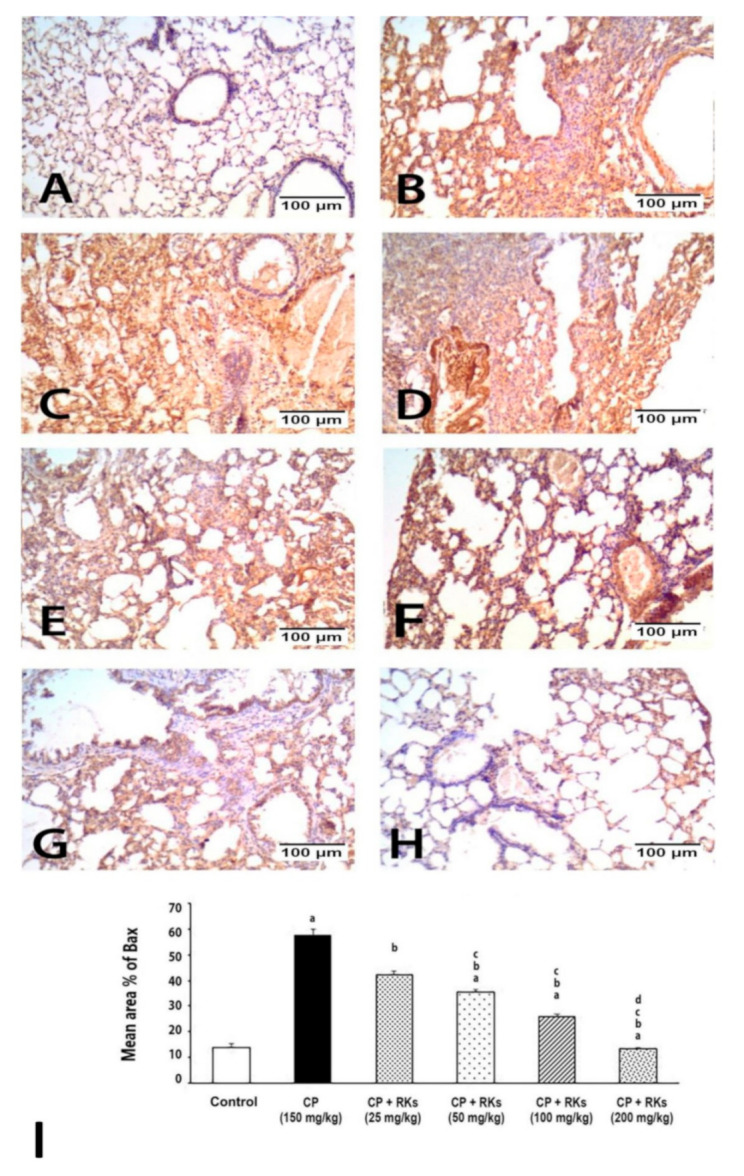
Photomicrographs showing Bax immunoexpression on the lung tissue of different experimental groups. (**A**) Negative to weakly immunoreactivity control group. (**B**–**D**) strong immunoreactivity of CP group. Immunoreactivity to Bax was decreased as the dose of RKs increased as shown in (**E**) RKs 25 mg/kg, (**F**) RKs 50 mg/kg, (**G**) RKs 100 mg/kg and (**H**) RKs 200 mg/kg. (**I**) graph of the mean area % of immunoreaction to Bax. Results are expressed as mean ± S.E. and analyzed using One Way ANOVA followed by Bonferroni’s post-hoc test at *p* ≤ 0.05 (*n* = 4–6). ^a^ Indicates significant differences from +control (Control) mice. ^b^ Indicates significant differences from CP, ^c^ Indicates significant differences from RKs (25 mg/kg/p.o), ^d^ Indicates significant different from RKs (50 mg/kg/p.o.).

Examination of the immunostained lung sections for detection of Bax in all experimental groups showed that the control group immunoreactivity was negative or faint. The most significant immunoreaction was observed in the CP group, which was reduced gradually as the dose intake increased in the treated groups as follows: RKs (25 mg/kg), (50 mg/kg), (100 mg/kg), and (200 mg/kg) (Figure 3).

**Figure 4:**
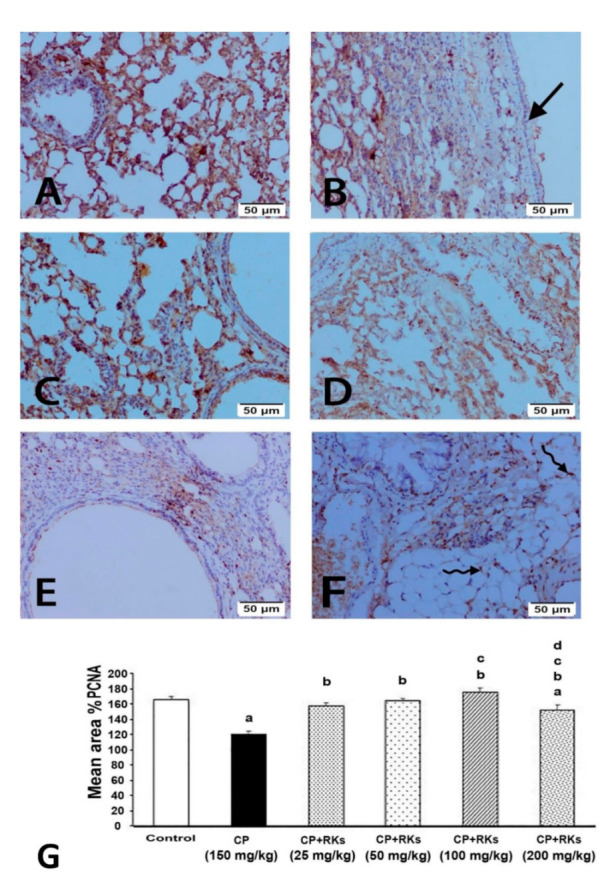
Photomicrographs showing PCNA immunoexpression on the lung tissue of different experimental groups. (**A**) Moderate immunoreactivity of control group. (**B**) Negative immunoreactivity of lining epithelium of bronchioles of CP group (arrow). Note that the treated groups with RKs (**C**) 25 mg/kg, (**D**) 50 mg/kg, (**E**) 100 mg/kg and (**F**) 200 mg/kg were positive; the most intense in the lining epithelium of alveoli of treated group with 200 mg/kg (curved arrow). (**G**) graph of the mean area % of immunoreaction to PCNA. Results are expressed as mean ± S.E. and analyzed using One Way ANOVA followed by Bonferroni’s post-hoc test at *p* ≤ 0.05 (*n* = 4–6). ^a^ Indicates significant differences from +control (Control) mice. ^b^ Indicates significant differences from CP, ^c^ Indicates significant differences from RKs (25 mg/kg/p.o), ^d^ Indicates significant different from RKs (50 mg/kg/p.o).

Examination of the immunostained lung sections for detection of PCNA in all experimental groups showed that the immunoreactivity was localized in the nuclei of the lining epithelium of the bronchioles and alveoli. The control group immunoreactivity was moderate, while in the CP group, the lining epithelium of the bronchioles was mainly negative, while those of the alveoli had a moderate reaction. Treated groups with RKs (25, 50, 100, and 200 mg/kg/p.o.) showed positive immunoreactivity in the lining epithelium of both the bronchioles and alveoli; the most intense immunoreactivity was at the lining of the alveoli of the treated group with RKs (200 mg/kg) (Figure 4).

### 3.3. Effect of CP and RKs on Lung Oxidative Stress Biomarkers

Table 1 depicts the change in some biochemical markers related to oxidative stress in lung homogenates. To examine the effect of CP on oxidative stress, MDA was assessed as a marker for damage in the lipid bilayer of a cell membrane. In mice acutely administered CP (150 mg/kg/i.p.), the MDA levels in lung tissues significantly increased. Pre-treatment with graded doses of RKs (25, 50, 100, and 200 mg/kg/p.o.) for two weeks resulted in a decrease in MDA levels in lung tissues in a dose-dependent manner.

The present study exhibited that the activities of catalase and SOD in lungs tissues were significantly reduced in CP-treated mice compared with the normal control. However, a marked rise in catalase and SOD activities was observed on administration of different doses of RKs prior to CP administration. Interestingly, RKs (200 mg/kg/p.o.) are capable of recovering SOD and catalase activities to similar levels of the control (Table 1). Thus, pre-treatment with RKs can protect lung tissues from the toxic effect of CP by combating free radicals evidenced by ameliorating the changes in biomarkers of oxidative stress.

### 3.4. Effect of CP and RKs on NF-KB Pathway

**Figure 5:**
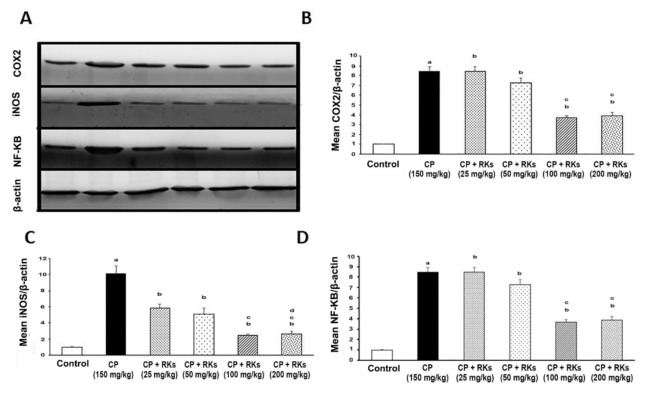
Western blot analysis of NF-KB, iNOS, and COX-2 expression in the lung tissues of male mice treated with RKs against CP. (**A**) The lung tissues from different experimental groups were lysed and the proteins separated by SDS–PAGE and western blotted using antisera against COX-2, iNOS, NF-KB and β-actin was used as a loading control. The bar graphs show quantitative relative levels of (**B**) COX-2 (**C**) iNOS, and, (**D**) NF-KB protein expression for Control, CP (150 mg/kg, i.p.), CP+RKs with different doses (25, 50, 100, and 200 mg/kg/p.o.). All values are presented as means ± SE (*n* = 4–6). Superscript symbols indicate a significant difference at *p* ≤ 0.05 using one- way ANOVA followed by the Bonferroni s test for multiple comparisons (*n* = 3). ^a^ Indicates significant differences from +control (Control) mice, ^b^ Indicates significant differences from CP, ^c^ Indicates significant differences from RKs (25 mg/kg/p.o.).

Figure 5 shows the effect of graded doses of RKs (25, 50, 100, and 200 mg/kg/p.o.) for 14 days on the expression of NF-KB, iNOS, and COX-2 in lung tissues. Image J software was used to determine the intensity of target proteins (NF-KB, iNOS, and COX-2) against control sample of β-actin by total protein normalization. Statistical analysis utilizing one-way ANOVA detected the oral administration of graded doses of RKs decreased the expression of NF-KB, iNOS, and COX-2 in a dose-dependent manner compared with the CP-treated group.

### 3.5. Effect of CP and RKs on Nuclear DNA Fragmentation

**Figure 6:**
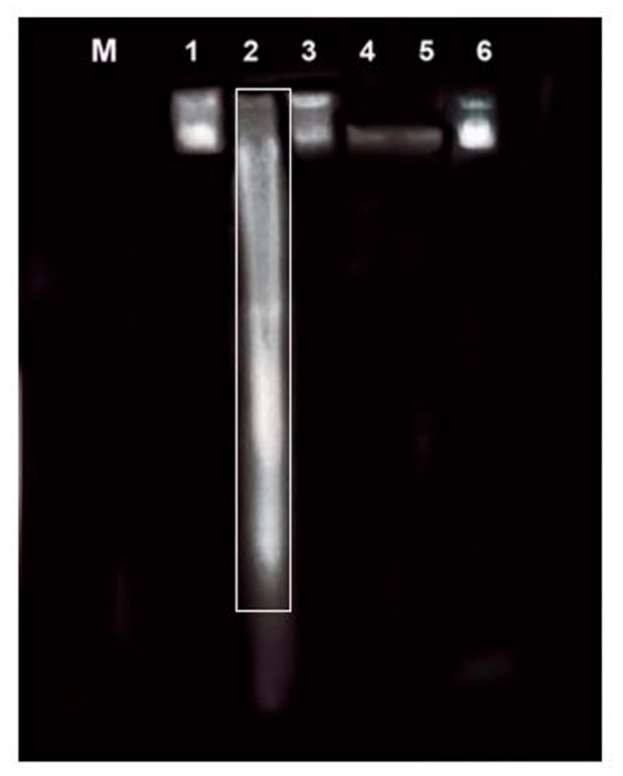
Effect of CP (150 mg/kg/i.p) and different doses of RKs (25, 50, 100, 200 mg/kg/p.o )on the integrity of nuclear DNA (nDNA) extracted from lung tissues of male mice (*n* = 3). Lane M, 100 kb DNA ladder; intact nDNA of Control (lane 1) and (lane 2) CP-treated mice, nDNA samples of RKs with different treated doses such as: RKs (25 mg/kg/p.o) of (lane 3), RKs (50 mg/kg/p.o) of (lane 4), RKs (100 mg/kg/p.o) of (lane 5), and finally RKs (200 mg/kg/p.o) of (lane 6) for 2 weeks before acute exposure to CP (150 mg/kg/p.o).

Figure 6 demonstrates the qualitative alterations in the integrity of the genomic DNA extracted from lung tissues of male mice. The results from agarose gel electrophoresis showed that, for nDNA isolated from untreated control (lane 1), RKs (lanes 3, 4, 5, and 6) exhibited total ladder and smear negativity. However, CP exposure resulted in a characteristic DNA ladder pattern with smearing in lung tissues as a marker of apoptosis. In addition, a dramatic oligonucleosome-length degradation of genomic DNA was observed (lane 2, Figure 6). These findings demonstrated that different doses of RKs (25, 50, 100, and 200 mg/kg/p.o.) abolished the ladder pattern of genomic DNA cleavage in the lung tissues of CP-treated mice.

## 4. Discussion

Pulmonary toxicity is a common side effect of CP that may limit its long-term administration in oncology settings. In the current study, pulmonary injury induced by CP is marked histologically by causing endothelial cell destruction, type I and type II alveolar epithelial cell damage, alveolar oedema, hemorrhage, and inflammatory cell infiltration. These histological changes suggest that exposure of lung tissues to CP can evoke inflammatory response with subsequent release of numerous inflammatory mediators. These mediators can result in the increase of ROS. In addition, CP metabolites could potentially cause overproduction of ROS [16,17]. This was evident in our study by the decrease in the activities of some antioxidant enzymes such as catalase and SOD, and the increase in lipid peroxidation (MDA) in the lungs of mice exposed acutely to CP. The rise in MDA levels reflects the damage occurring in the cell membrane of lung tissues. Lipid peroxidation enhances the breakdown of polyunsaturated fatty acids causing deterioration of the membrane function and increased tissue permeability. Aldehydes that are formed during the lipid peroxidation have been coupled with oxidative stress in tissues [18].

On the other hand, there was a marked decrease in some endogenous defensive antioxidant enzymes, which indicated marked oxidative stress upon CP administration. SOD detoxifies the superoxide anion by its conversion to H_2_O_2,_ and O_2_. Catalase converts the toxic H_2_O_2_ into water. This is further evidence that CP potentially induces oxidative stress and depletes the pulmonary antioxidant defensive enzymes. It was shown previously that CP increased the production of ROS including H_2_O_2_ in addition to the release of reactive nitrogen species (RNS), including nitric oxide (NO), peroxynitrite (ONOO-), and nitrogen dioxide [19]. RNS is implicated in the pathogenesis of lung disorders including infections, acute lung injury, and adult respiratory distress syndrome [20]. Moreover, CP was found to deplete the glutathione stores, which are considered as a second line of defence after enzymatic anti-oxidants [19].

Consistent with the oxidative stress status produced by CP, DNA damage and activation of apoptotic pathways were observed. The results of the DNA laddering assay showed a significant DNA fragmentation upon CP administration. Phosphoramide mustard and Acrolein are the active metabolites of CP that are responsible for reducing the growth of cancerous cells by acting at the DNA level [21]. This study demonstrated that CP produced acute DNA mutations and inhabitation of the ability of DNA to replicate, which produced fragmentation to DNA. In addition, the findings from the immunohistochemical study showed that lung tissues isolated from mice received CP and incubated with Bax monoclonal antibody demonstrated strongly positive staining reactivity. This suggests that CP activated the intrinsic pathway through regulation of the B-cell lymphoma 2 (BCL-2) family of proteins with subsequent up-regulation of the pro-apoptotic gene Bax. This is in agreement with Asiri’s study [22], which demonstrated that CP-induced apoptosis in the cardiac tissues of rat was mediated by intrinsic pathway. It has been shown that CP treatment resulted in an increase in p53 expression and Bax, and downregulation of the anti-apoptotic gene BCL-2 [22]. CP may inhibit DNA replication and cell proliferation, as evidenced by weakly positive PCNA staining reactivity of the nuclei of the lining epithelium of the bronchioles and alveoli.

NF-KB is an important transcription factor that is responsible for the up-regulation of several proinflammatory proteins. NF-KB plays a fundamental role in promoting the expression of macrophages by specific genes implicated in host defence, such as proinflammatory cytokines, nitric oxide synthase-2 (NOS-2), and COX-2 [23].

Acute administration of CP caused increased expression of some inflammatory markers including NF-ΚB and the pro-inflammatory biomarkers iNOS and COX2. The increase in inflammation triggers the action of the apoptotic pathway. CP was found to increase the inflammatory cytokines and the number of inflammatory cells such as eosinophils [24]. These findings were concomitant with the histological observation in the current study in CP-treated lung tissues, where there were significant increases in the cellular infiltrate and significant injury to the alveolar epithelium.

It is fascinating that RKs were shown to be a promising candidate in ameliorating pulmonary injury induced by CP. RKs improved the histological structures of lung tissues and reduced thickened alveolar septa, pulmonary congestion, and pulmonary parenchymal inflammatory cell infiltration following CP administration. One possible mechanism underlying the protective effect of RKs may be attributed to its antioxidant activity. The decrease in the MDA levels and increase in the activities of SOD and catalase upon RKs’ administration propose that it can mediate its action via modulation of the endogenous antioxidant defence system. Both SOD and catalase are considered as pulmonary scavengers to the free radicals generated from CP metabolites. Thus, the rise in their activities due to RKs contributes to the protection against CP-induced pulmonary injury. The antioxidant property of RKs was reported in several tissues including gastric [25], hepatic [14], and brain tissues [26].

The current study showed that different doses of RKs prevented the laddering pattern of DNA, and this is concomitant to the weak positively staining reactivity of the lung tissues upon incubations with Bax. Indeed, the immunohistochemical results of the pulmonary tissues treated with RKs for two weeks prior to CP administration revealed strongly positive PCNA staining reactivity, indicating an increase in the rate of turnover and cell renewal.

The reduction of oxidative stress status recoded by RKs pre-treatment was concomitant with a reduced level of inflammatory markers. RKs were reported previously to decrease inflammatory cytokines in non-alcoholic steatohepatitis [11] and in isoprenaline-induced myocardial infarction [27]. These findings were consistent with the histopathological results showing RKs-pre-treated lung tissues with much less inflammatory cells’ infiltration, oedema, and haemorrhage.

## 5. Conclusions

To conclude, RKs are a promising tool that can lessen the severity of pulmonary toxicity following CP therapy. They were able to reduce the pathological and biochemical changes occurring from CP administration. They can protect against lung injury through their antioxidant and anti-inflammatory properties. This study suggests that RKs could be used in oncology settings and in patients suffering from some autoimmune disease to protect against CP-induced lung toxicity. Future studies are required to investigate its clinical application.

## Figures and Tables

**Table 1:** Effect of different doses of Raspberry ketones (RKs) on some biochemical indicators of oxidative stress, namely MDA, Catalase and SOD activity, in lung tissue. Results are as expressed + S.E. *n* = 6 for each experimental group Superscript letters indicate a significant difference at *p* > 0.05 using one-way ANOVA followed by the Bonferroni’s test for multiple comparisons. ^a^ Indicates significant differences from control mice, ^b^ Indicates significant differences from CP.

	Control	CP (150 mg/kg/i.p.)	CP+RKs (25 mg/kg/day)	CP+RKs (50 mg/kg/day)	CP+RKs (100 mg/kg/day)	CP+RKs (200 mg/kg/day)
Catalase (U/mg protein)	105.87 ± 3.83	59.86 ± 4.72 ^a^	84.31 ± 4.75 ^b^	89.31 ± 1.70 ^b^	116.32 ± 12.25 ^b^	128.59 ± 9.30 ^b^
(SOD) (U/mg protein)	70.53 ± 1.87	40.51 ± 3.72 ^a^	55.32 ± 2.42 ^b^	56.28 ± 2.30 ^b^	68.36 ± 3.89 ^b^	74.59 ± 2.15 ^b^
(MDA) (nmol/mg protein)	322.22 ± 9.89	730.25 ± 16.07 ^a^	537.04 ± 13.73 ^b^	456.79 ± 5.12 ^b^	424.69 ± 6.6.7 ^b^	374.07 ± 5.32 ^b^
